# An inter-day assessment of the ABC parameters in the evaluation of progressive keratoconus

**DOI:** 10.1038/s41598-021-95503-8

**Published:** 2021-08-06

**Authors:** Ingemar Gustafsson, Tove Faxén, André Vicente, Anders Bergström, Anders Ivarsen, Jesper Østergaard Hjortdal

**Affiliations:** 1 Department of Clinical Sciences, Ophthalmology, Lund University, Skåne University Hospital, Kioskgatan 1, 222 85 Lund, Sweden; 2 Department of Ophthalmology, Aarhus University Hospital, Aarhus, Denmark

**Keywords:** Health care, Corneal diseases

## Abstract

The progression of keratoconus is commonly determined by comparing the results of corneal tomographic measurements on different occasions. However, investigations on the repeatability of measurements are commonly performed within the same day, thus not taking the inter-day variation into account. The effect of keratoconus disease severity on the measurement error is also seldom considered. In this post hoc investigation, the parameters A, B and C in the Belin ABCD Progression Display were evaluated in relation to disease severity in intra-day and inter-day measurements. Four consecutive measurements were performed on 61 patients with keratoconus on the same day (intra-day). In another cohort, four consecutive measurements were obtained and then repeated 3 days later in 25 patients with keratoconus and 25 healthy controls (inter-day). The results suggest that the diagnosis of disease progression would benefit from inter-day measurements, and the stratification of the parameters A and C according to disease severity. It is also recommended that tomographic systems such as the Pentacam HR be modified to allow the comparison of both single measurements and the mean of replicate measurements of the parameters used in the assessment of progression of keratoconus.

## Introduction

Keratoconus is the most common form of primary ectasia, and in cases of progressive disease, timely corneal crosslinking (CXL) can prevent further progression^1–3^. Although CXL was introduced in 2003^4^, the scientific evidence of its efficacy in halting continued progression was deemed to be of very low quality in a Cochrane Review in 2015^5^. The lack of robust evidence-based results also appears to have contributed to the seemingly late approval of CXL for the treatment of progressive keratoconus by the US FDA^6^. A serious drawback in scientific investigations on the effect of CXL in treating progressive keratoconus is that there is no consensus on the definition of progressive keratoconus nor adequate means of assessing treatment efficacy (i.e. treatment outcomes)^7^, which would facilitate the meta-analysis of data and accelerate the implementation of evidence-based treatment protocols.

The Belin ABCD Progression Display was recently developed with the aim of improving the diagnosis of progressive keratoconus, and is incorporated in the Pentacam HR tomography system^8^. The ABCD progression display assesses the anterior corneal curvature (A), the posterior corneal curvature (B) and corneal pachymetry at the thinnest point (C) in a 3 mm zone centred on the thinnest point. The visual acuity (D) can be added manually. The change in each of these parameters can be used to detect progression by making measurements over time. The software illustrates the changes graphically and calculates whether the change in the magnitude of a parameter (apart from D) exceeds the 80% or 95% one-tailed confidence interval, based on a reference population of subjects with keratoconus or healthy individuals. The latter population is suggested to be more representative of milder cases of keratoconus. It has been proposed that the ABCD progression display could be used to detect progression earlier than the commonly used maximum keratometry reading, K_max_^9^.

We have previously demonstrated an association between measurement error and the severity of keratoconus in measurements made with the Pentacam HR^10^. In that study, we suggested the stratification of detection limits for different parameters according to disease severity. This has also recently been proposed by other authors^11^. In a more recent study, we presented the inter-day repeatability of measurements in subjects with keratoconus and in healthy controls, using the Pentacam HR^12^. Apart from considering the effects on inter-day repeatability, the significant effects of disease severity were also elucidated. Furthermore, the difference between using single measurements and the mean of replicate measurements when assessing progression between visits was investigated. As the ABCD Progression Display is integrated into the Pentacam HR, which is the most commonly used tomographic instrument in the management of keratoconus^5^, it can be assumed that this software is widely used in both clinical practice and scientific investigations. It is therefore important to evaluate whether our previous findings are also relevant for the ABC parameters. It is of particular interest to analyse the inter-day effect on the ABC parameters, as these are based on intra-day measurements. Values of the ABC parameters obtained in our previous investigations were therefore analysed.

### Definitions and abbreviations

- Within-subject standard deviation (S_w_). The square root of the variance between subjects.
- Precision = 1.96 × S_w_. The difference between a measurement and the true value should lie below this limit in 95% of the measurements.
- Repeatability coefficient (RC) = 2.77 × S_w_. The difference between two measurements should lie below this limit in 95% of the pairs of observations.
- Coefficient of variation (CoV). S_w_ divided by the total subject mean.
- Intra-class correlation coefficient (ICC). The variance between subjects divided by [the variance between subjects + the variance within subjects].
- Prediction limit (PL) = 95% CI for differences between two future single measurements.
- A: Anterior curvature of the 3 mm zone over the thinnest point of the cornea.
- B: Posterior curvature of the 3 mm zone under the thinnest point of the cornea.
- C: Thickness at the thinnest point of the cornea (μm).

## Results

The ICC showed high values for all the measured parameters in all intra and inter-day measurements in all the groups. Therefore, variability could be interpreted as resulting from differences between subjects rather than within subjects (Tables 1, 2 and 3).

**Table 1:** Descriptive statistics and repeatability of Pentacam measurements made on a single day in subjects with keratoconus.

	Mean (SD)^a^	(Min–Max)^a^	S_w_ (95% CI)	RC (95% CI)	ICC (95% CI)	CoV (%)	Kendall’s tau-b^b^	P^b^ (2-tailed)	Spearman’s rho^b^	P^b^ (1-tailed)
**(n = 61)**										
A (mm)	7.02 (0.56)	(5.14–8.10)	0.067 (0.060–0.074)	0.18 (0.17–0.20)	0.99 (0.98–0.99)	0.95	− 0.162	0.07	− 0.246	0.03
B (mm)	5.37 (0.58)	(3.56–6.49)	0.087 (0.078–0.095)	0.24 (0.22–0.26)	0.98 (0.97–0.99)	1.61	− 0.031	0.73	− 0.035	0.39
C (μm)	485.2 (40.5)	(394.5–574.5)	5.47 (4.91–6.03)	15.2 (13.6–16.7)	0.98 (0.97–0.99)	1.13	0.174	0.05	0.265	0.02

A = Anterior curvature of the 3 mm zone over the thinnest point (mm). B = Posterior curvature of the 3 mm zone under the thinnest point (mm). C = Thickness of the thinnest point on the cornea (μm).

^a^Subject mean.

^b^Subject SD versus subject mean.

**Table 2:** Descriptive statistics and repeatability of intra-day Pentacam measurements in subjects with keratoconus and healthy controls.

	Mean (SD)^a^	(Min–Max)^a^	S_w_ (95% CI)	RC (95% CI)	ICC (95% CI)	CoV (%)		Kendall’s Tau-b^b^	_pb (2-tailed)_	Spearman’s rhob	pb (1-tailed)
**Intra-day measurements**											
Keratoconus patients (n = 25)											
A (mm)											
Day 0	7.18 (0.48)	(6.34–7.99)	0.054 (0.045–0.063)	0.15 (0.13–0.17)	0.99 (0.98–0.99)		0.78	− 0.284	0.047	− 0.396	0.025
Day 3	7.17 (0.49)	(6.34–7.90)	0.045 (0.037–0.052)	0.12 (0.10–0.14)	0.99 (0.98–1.00)		0.62	− 0.264	0.065	− 0.387	0.028
B (mm)											
Day 0	5.53 (0.51)	(4.68–6.45)	0.049 (0.041–0.056)	0.13 (0.11–0.16)	0.99 (0.98–1.00)		0.88	− 0.030	0.833	− 0.070	0.370
Day 3	5.52 (0.52)	(4.58–6.43)	0.054 (0.046–0.063)	0.15 (0.13–0.18)	0.99 (0.98–0.99)		0.99	− 0.193	0.176	− 0.312	0.065
C (μm)											
Day 0	492.6 (35.0)	(442.8–560.3)	3.85 (3.23–4.46)	10.7 (8.96–12.4)	0.99 (0.98–0.99)		0.78	− 0.087	0.543	− 0.116	0.290
Day 3	492.8 (35.3)	(437.5–561.3)	3.84 (3.23–4.46)	10.6 (8.94–12.4)	0.99 (0.98–0.99)		0.78	− 0.044	0.761	− 0.029	0.446
Healthy controls (n = 25)											
A (mm)											
Day 0	7.77 (0.23)	(7.34–8.20)	0.012 (0.010–0.013)	0.032 (0.027–0.037)	1.00 (1.00–1.00)		0.15	0.024	0.864	0.18	0.466
Day 3	7.77 (0.23)	(7.34–8.23)	0.013 (0.011–0.015)	0.035 (0.030–0.041)	1.00 (0.99–1.00)		0.16	− 0.78	0.590	− 0.076	0.358
B (mm)											
Day 0	6.31 (0.19)	(5.83–6.64)	0.044 (0.037–0.051)	0.12 (0.10–0.14)	0.95 (0.91–0.98)		0.69	0.230	0.107	0.34	0.048
Day 3	6.31 (0.20)	(5.83–6.66)	0.026 (0.022–0.030)	0.073 (0.061–0.084)	0.98 (0.97–0.99)		0.42	− 0.114	0.427	− 0.141	0.251
C (μm)											
Day 0	538.2 (23.0)	(493.0–580.3)	3.94 (3.31–4.57)	10.9 (9.17–12.7)	0.97 (0.95–0.99)		0.73	− 0.179	0.215	− 0.243	0.120
Day 3	539.4 (23.8)	(501.0–585.0)	4.13 (3.46–4.79)	11.4 (9.60–13.3)	0.97 (0.95–0.99)		0.76	− 0.120	0.400	− 0.212	0.154

A = Anterior curvature of the 3 mm zone over the thinnest point (mm). B = Posterior curvature of the 3 mm zone under the thinnest point (mm). C = Thickness of the thinnest point on the cornea (μm).

^a^Subject mean.

^b^Subject SD versus subject mean.

**Table 3:** Descriptive statistics and repeatability of inter-day Pentacam measurements in subjects with keratoconus and healthy controls (mean of replicates).

	Mean (SD)^a^	(Min–Max)^a^	S_w_ (95% CI)	RC (95% CI)	ICC (95% CI)	CoV (%)		Kendall’s Tau-b^b^	pb (2-tailed)	Spearman’s rhob	pb (1-tailed)
**Inter-day measurements**											
Keratoconus patients (n = 25)											
A (mm)											
All	7.17 (0.48)	(6.34–7.95)	0.046 (0.033–0.059)	0.13 (0.092–0.16)	0.99 (0.98–1.00)		0.64	− 0.377	0.009	− 0.481	0.007
< 7.33	6.81 (0.36)	(6.34–7.33)	0.060 (0.037–0.082)	0.17 (0.10–0.23)	0.97 (0.92–0.99)						
≥ 7.33	7.57 (0.20)	(7.34–7.95)	0.024 (0.015–0.034)	0.07 (0.040–0.094)	0.98 (0.95–1.00)						
B (mm)	5.52 (0.51)	(4.63–6.44)	0.044 (0.032–0.056)	0.12 (0.088–0.16)	0.99 (0.98–1.00)		0.79	0.113	0.427	0.132	0.265
C (μm)											
All	492.7 (35.1)	(442.3–560.8)	2.95 (2.13–3.77)	8.17 (5.91–10.4)	0.99 (0.98–1.00)		0.60	− 0.350	0.016	− 0.480	0.008
< 482.5	463.9 (12.9)	(442.3–482.4)	3.67 (2.20–5.13)	10.2 (6.10–14.2)	0.92 (0.76–0.98)						
≥ 482.5	519.3 (26.8)	(482.5–560.8)	2.07 (1.28–2.87)	5.75 (3.54–7.96)	0.99 (0.98–1.00)						
Healthy controls (n = 25)											
A (mm)	7.77 (0.23)	(7.34–8.21)	0.012 (0.009–0.015)	0.033 (0.024–0.042)	1.00 (0.99–1.00)		0.15	− 0.069	0.638	− 0.104	0.311
B (mm)	6.31 (0.20)	(5.83–6.65)	0.020 (0.015–0.026)	0.056 (0.041–0.072)	0.99 (0.98–1.00)		0.32	0.189	0.190	0.260	0.105
C (μm)	538.8 (23.4)	(497.0–582.6)	2.34 (1.69–2.98)	6.47 (4.68–8.27)	0.99 (0.98–1.00)		0.43	0.171	0.240	0.209	0.158

A = Anterior curvature of the 3 mm zone over the thinnest point (mm). B = Posterior curvature of the 3 mm zone under the thinnest point (mm). C = Thickness of the thinnest point on the cornea (μm).

^a^Subject mean.

^b^Subject SD versus subject mean.

### Repeatability and disease severity

**Figure 1:**
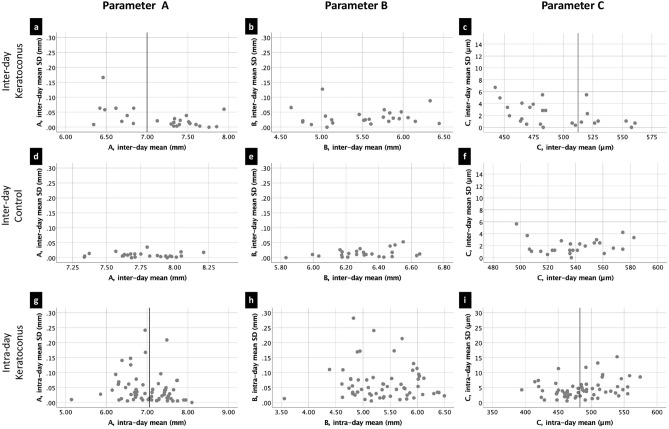
Mean values of inter-day and intra-day measurements of the parameters A, B and C, plotted against the standard deviation. (**a**) Inter-day measurements of parameter A in subjects with keratoconus, (**d**) inter-day measurements of A in control patients, (**b**) inter-day measurements of B in subjects with keratoconus, (**e**) inter-day measurements of B in the control group, (**c**) inter-day measurements of C in subjects with keratoconus and (**f**) inter-day measurements of C in the control group. Lower values of parameters A and B indicate more severe keratoconus, while lower values of C indicate less severe disease. The vertical lines indicate the median.

A correlation between the magnitude of a measured parameter and its SD indicates a worsening of the repeatability of the measurements with increasing parameter magnitude. Disease severity was found to be significantly associated with measurement error for the parameters A and C, but not for B (the correlation for the parameter A was not significant in the group n = 61 for two-tailed CIs but was significant for 1-tailed CIs). This correlation was more pronounced in inter-day measurements. One-tailed 95% CIs showed a stronger association than two-tailed 95% CIs (Table 3). The strongest association was seen in inter-day measurements of A in subjects with keratoconus (Spearman’s rho = − 0.481, p = 0.007, Kendall’s Tau-b = − 0.377, p = 0.009), followed by measurements of C in the same group (Spearman’s rho = − 0.480, p = 0.008, Kendall’s Tau-b = − 0.350, p = 0.02), and in C in the intra-day measurements in subjects with keratoconus (Spearman’s rho = − 0.265, p = 0.02, Kendall’s Tau-b = 0.174, p = 0.05) and in C in the same group (Spearman’s rho = − 0.265, p = 0.02, Kendall’s Tau-b = 0.174, p = 0.05). Nevertheless, in intra-day measurements of A in subjects with keratoconus the correlation was close to being significant (Spearman’s rho = − 0.246, p = 0.03, Kendall’s Tau-b = − 0.162, p = 0.07) (Tables 1 and 3). No significant association was found in measurements of B in subjects with keratoconus (Tables 1, 2 and 3). Neither was any significant association found between the repeatability and magnitude in the inter-day measurements of any of the parameters in the control group (Table 3). No significant association was found in intra-day measurements for all the parameters in both subjects with keratoconus and the control group with the exception of parameter A in day 0 and 3 in subjects with keratoconus (Spearman’s rho = − 0.396, p = 0.025, Kendall’s Tau-b = − 0.284, p = 0.047 and Spearman’s rho = − 0.387, p = 0.028, Kendall’s Tau-b = − 0.264, p = 0.065) and parameter B in day 0 in the control group (Spearman’s rho = 0.34, p = 0.048, Kendall’s Tau-b = 0.230, p = 0.107) (Table 2). Figure 1 illustrates the mean values for each parameter for inter-day and intra-day measurements in subjects with keratoconus and the healthy control group.

### Intra-day repeatability of measurements

In intra-day measurements in subjects with keratoconus, the best repeatability was found for parameter A, followed by C and B (Table 1) and the same happened when the repeatability in the intra-day measurements in day 0 and day 3 were evaluated in subjects with keratoconus (Table 2). In the control group, the repeatability in the intra-day measurements in day 0 and day 3 was clearly superior to the measurements in subjects with keratoconus (Table 2).

### Inter-day repeatability of measurements using a mean of replicates

The repeatability of inter-day measurements of the parameters A, B and C was better in the control group than in subjects with keratoconus (Table 3). It was a factor of 4 worse for A, a factor of 2 worse for B, and a factor or 1.2 worse for C in subjects with keratoconus.

The best repeatability in the inter-day measurements was seen in the control group for parameter A (RC = 0.033 mm, 95% CI 0.024–0.042 mm, CoV 0.15%), followed by B (RC = 0.056 mm, 95% CI 0.041–0.072 mm, CoV 0.32%) and C (RC = 6.47 μm, 95% CI 4.68–8.27 μm, CoV 0.43%). In subjects with keratoconus, the repeatability in the inter-day measurements was best for parameter C (RC = 8.17 μm, 95% CI 5.91–10.4 μm, CoV 0.60%), followed by A (RC = 0.13 mm, 95% CI 0.092–0.16 mm, CoV 0.64%) and B (RC = 0.12 mm, 95% CI 0.088–0.16 mm, CoV 0.79%). When stratifying parameter A, subjects with keratoconus with a value below the median value for that parameter (7.33 mm) showed a repeatability about 2 times better that those with a value above the median (RC = 0.017 mm, 95% CI 0.10–0.23 mm vs. RC = 0.007 mm, 95% CI 0.040–0.0943 mm). Repeatability was also approximately two times better when stratifying parameter C for subjects with keratoconus with a value of that parameter above the median value (482.5 μm) than for those with a value below the median (RC = 5.75 mm, 95% CI 3.54–7.96 mm vs. RC = 10.2 mm, 95% CI 6.10–14.2 mm) (Table 3).

### Inter-day repeatability of measurements using single measurements (PLs)

The PLs for single inter-day measurements in subjects with keratoconus were − 0.19 to 0.17 mm for parameter A, − 0.19 to 0.16 mm for B and − 12.5 to 12.9 μm for C. In the control group, the PLs for single inter-day measurements were − 0.04 to 0.05 mm for A, − 0.10 to 0.11 mm for B and − 10.6 to 12.9 μm for C (Table 4). When stratifying the parameters A and C according to the median value, the PLs for single inter-day measurements in subjects with keratoconus were − 0.25 to 0.22 mm for values of A below the median value, and − 0.11 to 0.081 mm for values above the median value, − 15.4 to 14.0 µm for values of C below the median value and − 9.51 to 11.5 µm for values above the median value (Table 4).

**Table 4:** Inter-day differences between single measurements of the parameters A, B and C with prediction limits for subjects with keratoconus and healthy controls (single measurements).

	Variance components			Mean difference	Lower prediction limit	Upper prediction limit
	${\widehat{\tau }}^{2}$	${\widehat{\sigma }}_{1}^{2}$	${\widehat{\sigma }}_{2}^{2}$	${\widehat{\alpha }}_{1}-{\widehat{\alpha }}_{2}$	${\widehat{\alpha }}_{1}-{\widehat{\alpha }}_{2}-2\times \sqrt{2{\widehat{\tau }}^{2}+{\widehat{\sigma }}_{1}^{2}+{\widehat{\sigma }}_{2}^{2}}$	${\widehat{\alpha }}_{1}-{\widehat{\alpha }}_{2}+2\times \sqrt{2{\widehat{\tau }}^{2}+{\widehat{\sigma }}_{1}^{2}+{\widehat{\sigma }}_{2}^{2}}$
**Subjects with keratoconus**						
A (mm)	0.0015	0.0020	0.0029	− 0.013	− 0.19	0.17
< 7.33	0.0027	0.0035	0.0046	− 0.013	− 0.25	0.22
≥ 7.33	0.00039	0.00035	0.0011	− 0.012	− 0.11	0.081
B (mm)	0.0011	0.0030	0.0024	− 0.019	− 0.19	0.16
C (μm)	5.34	14.8	14.8	0.18	− 12.5	12.9
< 482.5	10.2	17.7	16.1	− 0.69	− 15.4	14.0
≥ 482.5	0.94	12.0	13.6	0.98	− 9.51	11.5
**Healthy controls**						
A (mm)	0.0001	0.0002	0.0001	0.003	− 0.04	0.05
B (mm)	0.0001	0.0007	0.0019	0.003	− 0.10	0.11
C (μm)	0.90	17.02	15.51	1.17	− 10.6	12.9

A = Anterior curvature of the 3 mm zone over the thinnest point (mm). B = Posterior curvature of the 3 mm zone under the thinnest point (mm). C = Thickness at the thinnest point on the cornea (μm).

${\widehat{\tau }}^{2}$ = squared between-subject mean variance between Day 0 and Day 3; ${\widehat{\sigma }}_{1}^{2}$ = squared within-subject mean variance on Day 0; ${\widehat{\sigma }}_{2}^{2}$ = squared within-subject mean variance on Day 3;${\widehat{\alpha }}_{1}-{\widehat{\alpha }}_{2}$ = difference between means on Day 0 and Day 3.

### Inter-day progression

In a randomized comparison between two measurements in each subject with keratoconus, six subjects (24%) showed progression according to one parameter (in three of these subjects the parameter A indicated progression, while in two subjects the parameter B suggested progression), and in one subject both parameters A and B indicated progression. In a second randomized comparison among the subjects with keratoconus, progression was indicated by one parameter in two of the subjects (8%). In one of these subjects parameter A indicated progression, while in the other B suggested progression. Two parameters (A and B) indicated progression in three of the subjects (12%), and all three parameters suggested progression in one of the subjects (4.0%).

## Discussion

The results of this study demonstrate the statistically significant association between disease severity and measurement error in the parameters A and C, but not B, in the Belin ABCD progression display. This association was more pronounced in inter-day measurements than in intra-day measurements. One-tailed 95% CIs also showed a stronger association with disease severity than two-tailed 95% CIs. These findings suggest progression should be diagnosed based on limits stratified according to disease severity for the parameters A and C. There appears to be a threshold at 7.0 mm for A, i.e. approximately 48 D, at which the measurement error begins to increase. This threshold appears to be equivalent to that for K_max_, which is not surprising as they are based on the same measurements^12^. The association between measurement error and disease severity was statistically significant for both A (Kendall’s Tau-b = − 0.377, p = 0.009) and K_max_ (Kendall’s Tau-b = 0.483, p = 0.0001), although the association for A was somewhat weaker. As a lower value of A indicates greater disease severity, Kendall’s Tau-b is negative, whereas a lower value of K_max_ indicates less severe disease. The threshold for C is at approximately 500 µm, below which measurements are more prone to error. It was also reported in a recent study that the repeatability of measurements of A, B and C deteriorated with increasing disease severity^11^. However, those calculations were based on intra-day measurements, and the association between deteriorating repeatability and disease severity was not investigated per se. An inter-day scenario is more appropriate as this reflects the clinical situation. Factors such as changes in the shape of the cornea due to diurnal variation or the natural biomechanical weakness of corneas affected by keratoconus could lead also to deterioration in the repeatability of inter-day measurements. However, other factors may improve the repeatability of measurements, such as learning effects among the patients. No association was seen between the measurement error and the magnitude of the measured parameters among healthy controls, and the repeatability of these measurements was clearly superior to those obtained in patients with keratoconus, in particular regarding the parameters A and B.

Progression can be assessed in the ABCD progression display by comparing the results with the 80% or 95% CIs obtained from a reference cohort of patients with keratoconus, or from a reference cohort of healthy subjects. The latter could be appropriate in subjects with less severe keratoconus, as the repeatability of these measurements will probably be more similar to those in a healthy cohort than a general cohort of patients with all stages of disease. In fact, in the abovementioned study^11^ the repeatability of measurements of A, B and C was reported to be identical in healthy subjects and in subjects with subclinical keratoconus. However, there will be a threshold at which some subjects with keratoconus will be over-diagnosed as progressive if compared to a healthy cohort. If stratified limits were implemented in the detection of progression in keratoconus, there would be no need for a comparison with a healthy cohort.

As well as considering the effects of disease severity, the thresholds at which progression could be detected were evaluated assuming two clinical scenarios: using one measurement on each occasion, and using the mean of replicate measurements (in this case the mean of four). This has been addressed in a few studies^12–14^ but it is seldom considered in the enrolment of subjects in clinical studies on CXL, and there is no software in the Pentacam HR allowing for the comparison of mean values. In order to avoid unnecessarily narrow and erroneous prediction limits for single measurements, the variance between the four replicates was included in the statistical analysis^15^. This provided more accurate results and reduced the risk of over-interpreting the results as indicating progression. However, and as expected, it can be concluded that comparing the mean values obtained on each occasion further improves the ability to detect progression, and it is therefore recommended that appropriate software be developed for this purpose.

The ABCD progression display is based on one-tailed 80% and 95% CIs. On the one hand, one-tailed intervals seem logical, as only a decrease in the magnitude of the parameter indicates progression; but on the other, the parameters can increase or decrease, which suggests that two-tailed intervals are more appropriate. Two-tailed 95% CIs were used in this study, and 80% CIs were avoided. The 95% CIs of the non-stratified repeatability of measurements of the parameters A and B in this study were wider than those used in the ABCD progression display, suggesting that there is a risk of over-interpreting the results as indicating progression. Empirically, the proportion of false positive results in the inter-day scenario was 24% (n = 6), for one or more parameters. When this analysis was repeated the same results were obtained. This empirical analysis describes a one-to-one measurement scenario and the false positive results are explained by the fact that the 95% prediction limits (reflecting a one-to-one measurement scenario) are wider than the 95% CIs in the Belin ABCD progression display, in particular for parameters A and B. It is important to note that only subjects with Stage 1–2 AKC (Amsler Krumeich Classification System) were included in this inter-day analysis. If subjects with Stage 3 disease had also been included, this would most likely have increased the proportion of false positive results due to the association between measurement error and disease magnitude. However, if the means of replicates were compared between days this would, as expected, reduce the number of false positive progressions. Unfortunately, this feature is not available in the Pentacam HR and could thus not be tested empirically.

When stratifying the parameters A and C above/below the median value, those with more advanced disease showed an approximately two times poorer repeatability for both the single measurements and the mean of replicates than those with less advanced disease. If comparing the limit in the ABCD Progression Display with the results for subjects with more advanced disease (bearing in mind that the whole cohort consisted of subjects with less advanced keratoconus) there would have been a further shift towards false positive results. However, in the group with the lower disease severity, the repeatability was close to the limit in the ABCD Progression Display for the scenario involving single measurements. If, on the other hand, the mean of replicate measurements is used, the 95% CIs of the repeatability of measurements of parameters A and C are below the limit in the ABCD Progression Display, leading to the risk of false negative results. In this case, it appears reasonable to compare this group with the suggested limits for a normal population in the Belin ABCD Progression Display. While the limits for parameter C are rather similar, the repeatability of the measurements of parameter A is still three times higher in the below-median group of keratoconus than in the normal population in the ABCD Progression Display, highlighting the difference in the repeatability between healthy subjects and subjects with keratoconus. The subjects included in the below-median group had K_max_ values ranging from 44.8 to 48.6 D. The repeatability of the measurements in the healthy controls in this investigation was similar to that presented in the Belin ABCD Progression Display.

A possible weakness of this study is that the optimal time frame for comparing inter-day repeatability is unclear. We chose three days as we deemed this to be sufficient to allow for inter-day changes in corneal shape, but sufficiently short to avoid true disease progression. Males were overrepresented in the keratoconus groups, reflecting the gender difference in patients with keratoconus at our clinic^10^, and the healthy controls were not matched for sex or age. We believe that diurnal variation would not affect the measurements significantly. The measurements were in general obtained between 09.00 a.m. and 15.00 p.m. It has been suggested previously that the corneal thickness is significantly reduced within the first 1–2 h after awakening but then remains relatively unchanged during the daytime^16,17^. In fact, the diurnal variation of keratometric and corneal thickness measurements in subjects with keratoconus has been suggested to be clinically insignificant^18^ if obtained between 09.00 a.m. and 17.00 p.m. We therefore believe that the results in this investigation are applicable in a daytime setting.

There is no gold standard for measuring progress in keratoconus, and thus measurement accuracy is of paramount importance, in both clinical practice and scientific investigations. As mentioned in the introduction, there is no consensus on the definition of progression. However, a consensus on which parameters should be used may be less important than understanding the repeatability and the dynamics of the parameters used and designing the investigation accordingly. This would be an important step towards facilitating the meta-analysis of data. More specifically, the use of reference data in the Belin ABCD Progression Display based on inter-day measurements should be considered. The association between measurement error and disease severity should also be considered for parameters A and C as this would allow progression to be diagnosed earlier in patients with less severe disease, and help avoid erroneous diagnosis of progression in those with more advanced disease. Furthermore, it is desirable that tomographic systems such as the Pentacam HR allow for the comparison of both single measurements and the mean of replicates for parameters used in the assessment of progression of keratoconus.

The findings of this investigation could be of interest for developers of software for the detection of progression in keratoconus, but may also be useful in clinical practice. The results of measurements of the A, B and C parameters are presented in the Progression Display and changes in the magnitude of the parameters between visits can be evaluated by comparing with the results of this investigation. However, clinicians would probably find it more practical to compare single measurements between visits as the mean of replicates would have to be calculated manually, as the current system does not allow for the comparison of mean values.

## Subjects and methods

The studies were conducted at the Department of Ophthalmology at Skåne University Hospital, Lund, Sweden, according to the declaration of Helsinki. The Regional Ethics Committee in Lund, Sweden, approved the studies (No. 2015/373).

### Enrolment

Patients with keratoconus fulfilling the inclusion criteria described below were enrolled consecutively after signing an informed consent form. The inclusion criteria were: keratoconus Stage ≤ 3 (Investigation 1)^10^ and keratoconus Stage ≤ 2 (Investigation 2)^12^ with no history of, and no current signs of, other ocular pathology, including ocular surface disease and external diseases such as dry eyes and atopy. Only subjects who had not undergone prior ocular surgery and who were aged ≥ 18 years were recruited and pregnant and breastfeeding women were also excluded^10,12^. Contact lens wear was discontinued at least 2 weeks before the measurements were made^10,12^. Subjects with advanced keratoconus (Stage 4) were excluded from Investigation 1^10^ due to the presence of corneal scarring. In Investigation 2^12^, patients with Stage 3–4 keratoconus were excluded as the purpose was to study subjects with less advanced disease. In both investigation 1^10^ and 2^12^ keratoconus was diagnosed clinically and by examination using The Pentacam HR. More specifically, the sagittal curvature pattern, posterior and anterior elevation maps, and corneal thickness pattern were assessed, in addition to information from the Belin-Ambrosio Enhanced Ectasia Display.

Sixty-one patients (Investigation 1)^10^ and 25 patients (Investigation 2)^12^ were enrolled. Only one eye was eligible for inclusion in 31 subjects in these investigations due to previous CXL, previous penetrating keratoplasty or too advanced stage of keratoconus. If two eyes were eligible for inclusion, both were examined (see “Examination” below). Computerised randomisation was performed in subjects where both eyes met the inclusion criteria to select one eye for inclusion in the study (41 right eyes and 45 left eyes). Seventy-six participants were males, and 10 females, and the mean age of all participants was 28 years (18–45 years).

Healthy controls (Investigation 2)^12^ (n = 25) were enrolled from among medical students and residents in ophthalmology after signing an informed consent form. The inclusion criteria were: age ≥ 18 years, no history of any ocular pathology or previous ocular surgery. Pregnant and breastfeeding women were excluded. Ocular pathology was excluded by clinical examination and by examination using the Pentacam HR. Only one eye was eligible for inclusion in three patients, due to scarring of the cornea. If two eyes were eligible for inclusion, both were examined and computerized randomization was performed, as described above, resulting in 12 right eyes and 13 left eyes. Fourteen participants were males, and 11 females, and their mean age was 29 years (23–41 years).

### Instruments

The Pentacam HR is a Scheimpflug-based tomographic system (Pentacam HR, version 1.20r10, Oculus Optikgeräte GmbH, Wetzlar, Germany). The technical features of this system have been described elsewhere^19^. The default setting of 25 pictures/s was used.

### Examination

Measurements were made on a single day (Investigation 1)^10^ and on two separate occasions (Investigation 2)^12^ by the same examiner (IG). In the latter study, 4 consecutive measurements were made on Day 0, and four on Day 3. Subjects were instructed to blink between measurements, but not to lean back. Measurements were made during normal working hours without taking diurnal corneal variation into account. Only examinations deemed “OK” by the Pentacam were accepted. The right eye was examined first, then the left, if both eyes were eligible for inclusion. This represents normal clinical practice where both the patient’s eyes are usually examined. When recruitment to the study was complete, computerised randomisation was performed to select one participating eye per subject.

### Statistical methods and calculations

The values obtained from the four replicate measurements were used to calculate the repeatability in Investigation 1. The measurements obtained on Day 0 and Day 3 in Investigation 2 were averaged for each day, and used to calculate the inter-day repeatability in the clinical situation where the mean value of several measurements is used to assess progression. When calculating prediction limits in the clinical scenario where single measurements are used to assess progression, the variance between replicate measurements was included in the calculation to provide more accurate results.

IBM SPSS Statistics 22 for Windows (IBM Corporation, Armonk, NY, USA) and SAS Enterprise Guide 6.1 for Windows (SAS Institute Inc., Cary, NC, USA) were used for statistical analyses. Results were considered statistically significant when the p-value was ≤ 0.05. Descriptive statistics are given as subject mean, standard deviation (SD), and minimum and maximum values. Repeatability was assessed by calculating the within-subject SD, precision, repeatability coefficient, intra-class correlation and coefficient of variation with associated confidence intervals (CIs)^20–22^. Kendall’s Tau-b was used to assess the relationship between the mean and SD, and natural logarithm transformed data were analysed when appropriate. The limits of agreement (denoted prediction limits) were calculated including the variance of the replicates using a linear mixed-effect model^15^.

In the empirical analysis of progression, the four measurements in the inter*-*day data were randomised to define one measurement as the baseline (at Day 0), and the other as the follow-up measurement (at Day 3), for each subject. The procedure was repeated to confirm the results.

## Supplementary Information

Supplementary Information 1.

Supplementary Information 2.

Supplementary Information 3.

## Data Availability

All data are available as “Supplementary information”.
